# Gene Expression Analysis of nc-RNAs in Bipolar and Panic Disorders: A Pilot Study

**DOI:** 10.3390/genes14091778

**Published:** 2023-09-09

**Authors:** Fabrizio Bella, Maria Rosaria Anna Muscatello, Angela D’Ascola, Salvatore Campo

**Affiliations:** 1 Psychiatry Unit, Department of Clinical and Experimental Medicine, University of Catania, 95124 Catania, Italy; 2 Psychiatry Unit, Department of Biomedical Sciences and of Morphological and Functional Images, University of Messina, 98122 Messina, Italy; maria.muscatello@unime.it; 3 Molecular Biology Laboratory, Department of Clinical and Experimental Medicine, University of Messina, 98122 Messina, Italy; adascola@unime.it; 4 Molecular Biology Laboratory, Department of Biomedical Sciences and of Morphological and Functional Images, University of Messina, 98122 Messina, Italy; scampo@unime.it

**Keywords:** bipolar disorders, panic disorder, molecular genetics

## Abstract

Background: Bipolar Disorder (BD) is a chronic, highly disabling mood disorder. Among the major comorbidities, Panic Disorder (PD) is often associated with BD. This could suggest a common genetic and pathophysiological background between these two conditions, as suggested by previous studies. Despite the widespread diffusion of these conditions, little is still known about the exact pathophysiological dynamics that underlies them. Non-coding RNAs have recently started to gain attention in psychiatry research, with several papers indicating the dysregulation of lncRNAs as a possible key factor in etiopathogenesis of several mental disorders. In the light of the above, the aim of this study is to evaluate the gene expression levels of *MALAT1*, *PANDA*, *GAS5*, *HOTAIR* lncRNAs and *miR-221-5p* microRNA, which are highly expressed in the CNS, in drug-naïve/drug-free bipolar and panic patients. Methods: the experimental plan envisaged the recruitment of sixteen patients with a first diagnosis of type one or type two BD and ten patients with PD. Patients with medical and/or psychiatric comorbidities were excluded. Peripheral venous blood was collected both from patients and healthy controls. Each of the patients recruited for the study was prescribed with therapy. Serum ncRNAs levels were remeasured after 5 months of therapy. Results: *MALAT-1*, *GAS-5* and *miR-221-5p* are significantly up-regulated in BD after therapy, while PD group showed a down-regulation of all the ncRNAs investigated after therapy. Conclusions: gene expression levels of the ncRNAs *miR-221*, *MALAT1*, *GAS5*, which are implicated in inhibitory modulation of the glucocorticoid receptor, are significantly over-expressed in bipolar patients following therapy, while all ncRNAs are significantly over-expressed in the PD T1 patients group compared with healthy controls. Data concerning PD represent, to our knowledge, a novelty.

## 1. Introduction

The majority of the human genome consists of genes that do not code for proteins. In fact, less than 3% of gene sequences are transcribed into messenger RNAs. Non-coding RNAs (nc-RNAs) represent a large and functionally heterogeneous class of the transcriptome in eukaryotes [1]. In recent years, many of these biomolecules have been structurally and functionally characterized, and for some of them, a fundamental role in the intricate processes relating to cell development and homeostasis has emerged [2]. Furthermore, data from the literature testify the importance of nc-RNAs, highlighting how their dysregulation contributes to the development of various pathological conditions, including some neuropshychiatric disorders, by altering the physiological processes of gene expression modulation [3]. For classification purposes, nc-RNAs can be distinguished on the basis of the number of nucleotides they comprise into two main families: short non-coding RNAs (sncRNAs), and long non-coding RNAs (lncRNAs). The former, whose length varies from a minimum of 20 nucleotides (e.g., micro-RNAs, miRNAs) up to a maximum of 200 nucleotides (e.g., small nucleolar RNAs, snoRNAs), have a particular role in the regulation of gene expression at post-transcriptional level [4], while the lncRNAs family includes molecules of over 200 nucleotides, transcribed by both RNA polymerase II (RNAPII) and RNA polymerase III (RNAPIII) [2,5], and whose role is encoded both in the nuclear environment, where they modulate gene expression at the transcriptional and epigenetic level, and in cytoplasm, where they act as regulators at the post-transcriptional level [6]. In the mammalian encephalon, a vast number of ncRNAs are expressed with high topographic selectivity and in a cell-specific manner, and the role of several ncRNAs in neurogenesis and synaptic plasticity is well established. Several papers point to ncRNAs, in particular the aberrant expression of miRNAs and lncRNAs, as possible key elements in the etiopathogenesis and pathophysiology of numerous neurodegenerative conditions, as well as some of the major morbid conditions of psychiatric interest, including schizophrenia, bipolar disorders and major depressive disorder [7]. Among the most studied psychopathologies in this respect, bipolar spectrum disorders (BDs) represent one of the entity of greatest clinical and social relevance, considering the lifetime prevalence of 2.4%, with an average age of onset of ~20 years and a suicide risk 20 times higher compared to the general population [8]. Furthermore, BD is among the mental illnesses with the highest rate of comorbidity with other psychopathological conditions. Among the major comorbidities of BD, anxiety disorders occupy a prominent position, with a lifetime prevalence in bipolar patients of between 11 and 79.2%. The coexistence of an anxiety disorder considerably complicates the clinical and therapeutic implications of BD as it can aggravate its course, masking its characteristic cyclic expressiveness, as well as interfere with treatment, leading to decreased responsiveness. Among anxiety disorders, panic disorder (PD) is so frequent among bipolar patients that some authors have hypothesized a common genetic diathesis [9]. Based on the above and in relation to the literature, this study aims to verify the gene expression levels of *miR-221-5p* and *DISC2*, *PANDA*, *HOTAIR*, *MALAT1*, *GAS5* lncRNAs in two groups of BD and PD patients. These nc-RNAs are richly expressed in the human central nervous system, where they modulate the processes of synaptogenesis, apoptosis and stress response.

## 2. Materials and Methods

### 2.1. Patients and Healthy Controls

The experimental plan involved the recruitment of patients with a first diagnosis of BD or PD and, therefore, had never received treatment for these conditions (drug-naive), or patients already known to have a diagnosis of BD or PD but had not been treated for at least six months (drug-free). All patients recruited for the study underwent a specialist medical examination at the psychiatry unit of the ‘Gaetano Martino’ university hospital in Messina. For each of the patients, the diagnosis of bipolar disorder or panic disorder was formulated by a psychiatrist according to the diagnostic criteria indicated by the DSM-V. The inclusion criteria were as follows: (1) age range from 18 to 55 years; (2) consistency between the clinical-symptomatological presentation and the criteria for making a diagnosis of bipolar disorder or panic disorder; (3) no psychopharmacological treatment in place for the past 6 months; (4) no hormone replacement therapy in place; (5) no relevant comorbidities in place (absence of autoimmune/allergological pathologies, absence of pathologies of internistic interest); (6) absence of relevant psychopathological comorbidities. From the eligible patients (BD n = 16; PD n = 10), 5 mL of peripheral venous blood was taken in EDTA tubes after their acceptance and signing of the informed consent form. The collection of samples was performed by the nursing staff of the psychiatry unit. Every sample was taken from the waste of basic haematochemical and haemochromocytometric investigations, which are routinely performed during hospitalization. The tubes were immediately centrifuged at 1200 rpm for 15 min at 4 °C. Centrifugation resulted in the separation of the corpuscular phase from the serum, which was collected and stored at −70 °C. A similar procedure was performed for the subjects in the control group. The blood samples from the controls were taken from the waste of blood donation samples. These subjects were assessed as conforming to be part of the healthy control group after specialist medical evaluation at the psychiatry unit of the university hospital “Gaetano Martino” in Messina. Each of the candidate subjects were administered a Hamilton depression rating scale (HAM-D) questionnaires to rule out recent or current states of depression, the Hamilton anxiety rating scale (HAM-A) to rule out recent or current states of anxiety, and the Mania rating scale (MRS) to rule out recent or current states of mania/hypomania. Each of the eligible subjects (CTRL n = 20) scored <8 on the HAM-D, <9 on the HAM-A and <11 on the MRS. In addition, each of the subjects in the CTRL group were included on the basis of the following criteria: (1) age range between 18 and 55 years; (2) total lack of evidence supporting a diagnosis of bipolar disorder or panic disorder from a clinical symptomatic perspective; (3) no recent stressful life events or substance abuse use in the last 6 months; (4) no current antibiotic, immunosuppressive/anti-inflammatory or hormone replacement therapy; (5) no history of psychopathologies; (6) no recent or current history of immunological/allergological pathologies, nor pathologies of internistic interest.

### 2.2. Therapy and Second Sampling 

For each of the patients recruited for the study was prescribed a therapy. Of the 16 patients in the BD group, 9 patients came to observation in the manic phase, and were therefore prescribed therapy with mood stabilizers (lithium sulphate 83 mg) and second-generation antipsychotics (olanzapine 10 mg). The remaining 7 in the BD group presented during a major depressive episode. In this subgroup, lamotrigine 50 mg, an SSRI (sertraline 50 mg/fluoxetine 20 mg) and a second-generation antipsychotic (olanzapine 5 mg/quetiapine 25 mg) were prescribed. All 10 patients in the PD group took fluoxetine 20 mg. All patients were discharged with a commitment to take a second sample at least five months after the first one. As performed similarly for the T1 sampling, serum was obtained by centrifugation of whole blood. 

### 2.3. RNA Extraction and cDNA Synthesis

Total RNA was extracted from 400 μL of each of the serum samples by using RNAzol (Genecopoeia, Rockville, MD, USA), following the kit protocol directions. After adding 1 mL of RNAzol (lncRNA extraction), the samples were left for 5 min at room T°. Subsequently, the samples were centrifuged at 12,000 rpm for 15 min. Following centrifugation, an aqueous phase was obtained, which was subsequently transferred into new tubes. RNA precipitation was achieved following the addition of 1 mL of isopropanol. After a 10 min incubation on ice, the samples were centrifuged at 12,000 rpm for 11 min. The obtained pellets were washed with 75 percent ethanol and again centrifuged at 7600 rpm for 5 min. The same step was repeated further. After leaving the tubes to dry under a fume hood for 5 min, the RNAs were resuspended in nuclease-free H_2_O, and their concentration (ng/μL) and purity (OD260/OD280 ratio) were assessed by NanoDrop spectrophotometer. To accomplish retrotranscription of mature miRNAs, complementary DNA (cDNA) was obtained by retrotranscription with the All-in-One miRNA qRT-PCR Detection Kit 2.0 from GeneCopoeia, which includes Poly A polymerase to elongate RNA nucleotide sequences with poly(A) tails and an oligodT adaptor primer that serves as a primer for retrotranscription. In contrast, the High-capacity cDNA Reverse Transcription kit from Applied Biosystem (Waltham, MA, USA) was used for lncRNAs. Both procedures were performed following the directions given in the protocols of the respective kits.

### 2.4. Real Time Polymerase Chain Reaction (rt-PCR) Analysis

The cDNAs obtained as a result of the retrotranscription were used to analyze the expression levels of *DISC2*, *PANDA*, *HOTAIR*, *MALAT1* and *GAS5* lncRNAs and *miR221*, using Applied Biosystem’s 7500 Real-Time PCR System instrument, employing PowerUp SYBR Green Master Mix (Applied Biosystem, Waltham, MA, USA) for lncRNA amplification and quantification. For miRNA 221-5p, the All-in-One miRNA qRT-PCR Detection Kit 2.0 was employed, which also employs SYBR Green chemistry and makes use of the Universal Adapter Primer that recognizes the oligo dT adaptor primer utilized in retrotranscription. The sequences of the specific primers used to amplify the lncRNAs and miRNA are shown in Table 1. To normalize the expression levels of miRNA in serum, *miR-39* was used as a spike-in (external control) (Norgen Biotech, Thorold, ON, Canada), in accordance with the literature [10]. For lncRNAs, *GAPDH* was used as endogenous control in accordance with the literature [11]. After the RealTime-PCR reactions, the threshold cycle (Treshold Cycle, Ct) was taken as a benchmark for the gene expression levels of target and housekeeping genes. Each of the samples was analyzed in duplicate. The expression levels of each of the samples was quantified according to the delta-delta CT (2−ΔΔCT) method. Initially, the average between the two samples was calculated for each target gene and each control. Next, delta CT (ΔCT) was calculated through the formula ΔCT = average CT (target) − average CT (housekeeping). After normalization, the ncRNAs levels were quantified according to the 2−ΔΔCT algorithm (relative analysis), the mean value of CTRL group target levels was chosen as the calibrator and the results are expressed according to the 2^−ΔΔCt^ calculation, as relative fold change.

ΔΔCT = ΔCt(Sample) − ΔCt(Calibrator).

### 2.5. Statistical Analysis

Data were expressed as mean values ± S.D. All assays were repeated three times to ensure reproducibility. For statistical analysis, the unpaired *t*-test with Welch’s correction (for independent samples) was used to compare t1 values with healthy controls and the paired *t*-test was used to compare t2 values with t1 values. Statistical significance was set at *p* < 0.05. Graphs were made using GraphPad Prism (version 7.04 for Windows), using a base-2 logarithmic scale.

## 3. Results

### 3.1. ncRNAs Expression in BD

**Figure 1:**
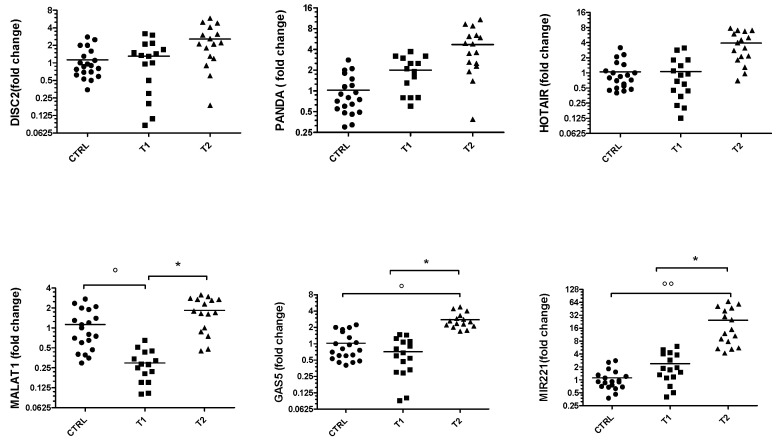
Gene expression of *DISC2*, *PANDA*, *HOTAIR*, *MALAT1*, *GAS5* and *miR-221* in BD group. Values are expressed as the fold change with respect to the control group ° *p* < 0.05 and °° *p* < 0.001 versus CTRL; * *p* < 0.05 versus T1.

Figure 1 shows the results of RT-PCR performed to evaluate the gene expression of the lncRNAs *DISC2*, *PANDA*, *HOTAIR*, *MALAT1* and *GAS5* and the *miRNA 221-5p* in BD patients at the time of first diagnosis (T1) and after at least 5 months of pharmacological therapy (T2). As can be seen from the graphs, the levels of all ncRNAs are higher in the T2 group, although the statistical significance is only appreciable for *MALAT-1*, *GAS-5* and *miR-221-5p*. These values were also compared with those obtained in healthy control group and it was found that *MALAT-1* is significantly down-expressed in the T1 subjects.

### 3.2. ncRNAs Expression in PD

**Figure 2:**
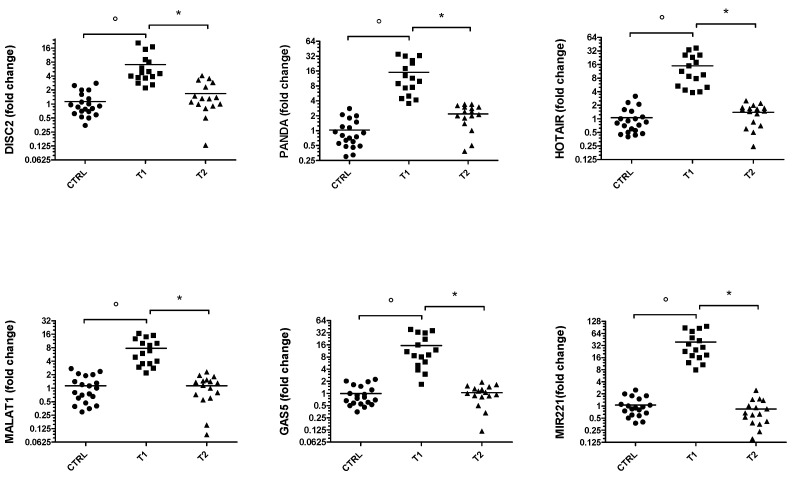
Gene expression of *DISC2*, *PANDA*, *HOTAIR*, *MALAT1*, *GAS5* and *miR-221* in PD group. Values are expressed as the fold change with respect to the control group. ° *p* < 0.001 versus CTRL; * *p* < 0.05 versus T1.

Similarly, the expression of the same ncRNAs was assessed in drug-naive PD patients (T1) after at least 5 months of therapy (T2) (Figure 2). The results are particularly interesting because all ncRNAs analyzed are significantly up-regulated in the T1 group patients compared with healthy controls. Furthermore, following therapy it can be seen that the gene expression levels of these ncRNAs are lowered compared to controls.

## 4. Discussion

Several data in the literature point to the dysregulation of certain ncRNAs as key elements in the CNS homeostatic perturbations underlying bipolar disorder. In the present study, gene expression levels of the ncRNAs *DISC2*, *PANDA*, *HOTAIR*, *GAS5*, *MALAT1*, and *miR-221-5p*, which are physiologically implicated in numerous processes of neuronal and glial metabolism, were evaluated. The analysis was conducted on serum samples from 16 drug-naive/drug-free patients with BD, taking one sample from the acute group (T1) and another after at least 5 months of therapy (T2). Given the frequent association between BD and anxiety disorders, gene expression levels of the same ncRNAs were assessed in a group of 10 drug-naive/drug-free patients with panic disorder, again performing one sampling in acute (T1) and another after at least 5 months of therapy (T2). These values were compared with those of 20 healthy controls. The results show that lncRNA *MALAT1* levels are significantly upregulated in BD T1 patient group compared with healthy controls (*p* < 0.05). Following 5 months of therapy (T2), *MALAT1* expression values are found to be similar to the controls group (*p* < 0.05). This result is in agreement with data already existing in the literature. *MALAT1* is highly expressed at the CNS level, where it modulates the expression of synaptogenesis-related genes through recruitment of SR family splicing proteins. In fact, synaptic density levels are found to be decreased in *MALAT1* gene knockdown models [12]. For *GAS5* lncRNA, there appears to be significant over-expression in the BD T2 patient group compared with the acute stages of the disease (T1) (*p* < 0.05). Down-regulation of *GAS5* in bipolar disorder compared with healthy controls is reported in the literature [13], and the trend shown in our study is in agreement with the data already collected by the authors. Interestingly, this lncRNA acts as a glucocorticoid receptor inhibitor [14], and its increase following therapy, as reported by our data, corroborates the hypothesis that increased glucocorticoid levels underlie neuronal and glial dysfunction in BD [15]. Similar results were obtained by comparing the gene expression levels of *miR-221* in the BD T1 group with those in the BD T2 group: miRNA is largely up-regulated in the group of bipolar patients undergoing therapy (*p* < 0.05). Similar results have been obtained by other authors, supporting the hypothesis of a correlation between *miR-221* levels and the response to lithium treatment [16]. In contrast, for *DISC2*, *PANDA* and *HOTAIR* lncRNA expression levels, no statistically significant difference was shown between the control group, BD T1 group and BD T2 group. This result is probably influenced by the low sample size. Regarding panic disorder, data from our study indicates that the levels of *DISC2*, *PANDA*, *HOTAIR*, *MALAT1* and *GAS5* lncRNAs are over-expressed in the T1 patient group, while the T2 patients show similar levels to the controls. The expression of *miR-221* is also similarly over-regulated. These findings, which could reflect metabolic perturbations in the CNS of PD patients, represent a novelty to our knowledge, as the above ncRNAs had not yet been investigated in panic disorder.

## 5. Conclusions

Our study showed that the gene expression levels of the ncRNAs *MALAT1*, *GAS5* and *miR-221-5p* are significantly over-expressed in bipolar patients following therapy. For *GAS5* lncRNA, implicated in inhibitory modulation of the glucocorticoid receptor, a significant increase in expression levels is observed following therapy in bipolar subjects; this finding represents a novelty compared to what has been documented in the literature. Further novelty concerns the gene expression levels of ncRNAs in Panic Disorder patients: from our observations, it was found that the ncRNAs *DISC2*, *GAS5*, *HOTAIR*, *MALAT1*, *PANDA* and *miR-221-5p* are significantly over-expressed in the T1 patients group compared with healthy controls. This over-expression is significantly modified by antidepressant therapy, until it reaches values similar to those in the control group. At present, the results of this study constitute preliminary data, which suggests that by increasing the number of samples and the number of genes investigated, it will be possible to elaborate broader considerations related to the search for biomarkers useful for the diagnosis and monitoring of the effectiveness of therapies for the disorders under investigation, as well as for a better understanding of the neurophysiopathological dynamics involved in the pathogenesis of BD and PD.

## Figures and Tables

**Table 1:** Sequences of primers.

Gene	Sequences
*GAS5*	F:CATTGGCACACAGGCATTAG R:TACCCAAGCAAGTCATCCATG
*DISC2*	F:CAGCCTCCCAAGTAGCTAGGAT R:CTGTAATCCCAGCACTTTGGAA
*PANDA*	F: GCCTGTTCCTCAATCCAAGA R: TTGCTTCTGGGCAGAACTTG
*MALAT1*	F: GGAAAGCGAGTGGTTGGTAA R:ATCCCTTTACACCTCAGTACGA
*HOTAIR*	F: GCACTCACAGACAGAGGTTTA R:CTCTGTACTCCCGTTCCCTAGA
*GADPH*	F: CACCAGGGCTGCTTTTAACTCT R: ATCTCGCTCCTGGAAGATGGT
*miR-221-5p*	F: TGAACATCCAGGTCTGGG

## Data Availability

Not applicable.
